# Is Prick of Conscience Associated With the Sensation of Physical Prick?

**DOI:** 10.3389/fpsyg.2020.00283

**Published:** 2020-02-21

**Authors:** Xyle Ku, Jonghwan Lee, Hyunyup Lee

**Affiliations:** Department of Psychology, Korea Military Academy, Seoul, South Korea

**Keywords:** prick of conscience, needle prick, moral judgment, metaphor, embodied cognition

## Abstract

“Prick of conscience” is a phrase to express feelings of guilt in both English and Korean. Particularly in South Korea, guilt is metaphorically associated with a sense of touch by pricking. Koreans commonly express feelings of guilt by using the metaphor, “It pricks my conscience.” Across three studies, we examined whether prick of conscience (i.e., feelings of guilt) is grounded in bodily experiences of physical prick (e.g., a needle prick), using a sample of Koreans. Participants who recalled past unethical acts were less likely to choose a needle prick rather than medication as a treatment for indigestion, whereas those who recalled ethical acts presented no significant difference in their willingness to receive either treatment (Study 1). Participants who decided to lie sensed the finger prick deeper and felt more pain as compared to those in the truth group or the control group (Study 2). Lastly, participants who had the finger prick rendered harsher moral judgments than participants in the control condition (Study 3). In line with an embodied cognition framework, these findings suggest that prick of conscience is not just a linguistic metaphor but can be embodied as physical sensations in forms of pricking.

## Introduction

*Prick of Conscience* is regarded as one of the most popular English poems of the Middle Ages (Lewis and McIntosh, 1982). The poem characterized by humility and fear describes divine-human relationships (Morey, 2012), and addresses a concern for confession of sins in the late medieval England (Galloway, 2009). The English word “prick” metaphorically illustrates disturbed feelings after engaging in guilty acts, as noticed in the metaphor, “prick of conscience,” which expresses feelings of guilt (e.g., Longman Dictionary of Contemporary English, Merriam-Wester Dictionary). People may express their remorse and penitence by saying, “I feel a prick of conscience,” or “It pricks my conscience.” Yet, the definition of “prick” is to physically pierce or puncture the object with a tiny sharp material (e.g., “A finger is pricked with a needle.”). This paper investigates a possible relationship between physical prick and emotional prick (i.e., guilt).

Notably, a similar expression exists in Korean. When a native speaker of Korean feels guilty about moral issues such as lying, he or she would say, “It pricks my conscience (or heart).” This metaphorical expression can be used both colloquially and formally in Korean. The usage of this Korean phrase can be found in the National Korean Dictionary in the entry for “prick”: “It pricks my conscience.” In this example, the verb “prick” refers to not only being physically punctured by sharp objects but also being emotionally disturbed by someone or at an event. In this sense, Kim (2005) stated that the feeling of guilt was “to be pricked” in Korean, which posits the possibility that this term can describe emotions and sensations. Earlier studies on Korean idioms also suggested that the phrase, “one’s heart is pricked,” describes an emotional experience of guilt (Chang and Chang, 1994; Park, 2002). Given that people commonly relate immoral conscience with acute pain from pricking, it raises an interesting question if feelings of guilt can be experienced in the physical setting (i.e., a needle prick) as reflected in the embodied cognition framework.

Research on embodied cognition assumes that bodily states are the consequences of social cognition as well as the causes of cognition (Barsalou, 2008). Furthermore, the extant literature in embodied cognition suggests that cognitive representations are grounded in the brain’s sensorimotor systems (i.e., sensation and action of the body; Niedenthal et al., 2005; Barsalou, 2010). Notably, given the nature of metaphorical expressions that delivers beyond the literal meaning (Landau et al., 2010; Lakoff, 2012), many scholars support the argument that there is a significant association between bodily experience and emotional experience in, for example, “coldness” (Zhong and Leonardelli, 2008), “comfort food” (Troisi and Gabriel, 2011), “fishiness” (Lee et al., 2015), “heavy-heartedness” (Min and Choi, 2016), “highness” (Schubert, 2005), “warmth” (Williams and Bargh, 2008), and “weight” (Jostmann et al., 2009). Taken together, these findings propose an intriguing possibility that the metaphorical sense of pricking as in the expression “prick of conscience” may be associated with physical prick (e.g., a needle prick).

Many research studies on guilty conscience have adopted the embodied cognition perspective. For instance, Zhong and Liljenquist (2006) demonstrated links between physical cleansing and moral cleanliness. They tested a metaphorical expression, “washing away one’s sins” and found that threatening one’s moral purity increases a desire to engage in physical cleansing behaviors. Moreover, results also indicated that the act of washing hands restores the sense of moral purity in participants. Similarly, researchers have demonstrated that physical cleansing alleviates guilt and reduces compensatory helping behaviors (Zhong and Liljenquist, 2006; Xu et al., 2014), enhances optimism (Kaspar, 2013), and moderates the impairing effect of threatened morality on the executive control system (Kalanthroff et al., 2017). Furthermore, priming feelings of guilt by manipulating participants’ body postures elevated negative backlash and increased pro-social behaviors (Rotella and Richeson, 2013). To sum up, previous studies on embodied cognition concerning conscience and guilt have primarily focused on the relationship between guilt and moral compensatory behaviors that attenuate feelings of guilt (e.g., physical cleansing, pro-social behaviors). However, less literature addresses the physical experiences accompanied by feelings of guilt.

Some major studies compare the bodily experience of guilt to a subjective feeling of weight. For example, Day and Bobocel (2013) asserted that the emotional experience of guilt could be embodied as a sensation of weight, as reflected in the metaphorical expression, “weight on one’s conscience.” Kouchaki et al. (2014) also found a significant association between subjective body weight and feelings of guilt. The results of this study indicated that individuals who wore a heavy backpack reported increased feelings of guilt as compared to those who wore a light backpack. Considering the association between guilt and subjective body weight based on the “weight of guilt” metaphor, we predict a similar relationship between the experiences of guilt and the bodily experience of the physical prick, inspired by the metaphorical expression of “prick of conscience.”

On a similar note, it is worth noting Bastian et al. (2011)’s study of guilt and pain. In their study, participants were asked to recall either personal unethical acts or casual interactions with people on the day before the experiment. They were then asked to put their hands in a bucket full of ice. Results indicated that participants who recalled guilty experiences in the past rated the experience as more painful than the control group. Accordingly, given that threatened moral self-image tends to heighten the sensitivity to physical pain, it is safe to assume that depending on feelings of guilt, individuals may report different degrees of sensitivity against finger prick.

As mentioned above, Zhong and Liljenquist (2006) found that threatening one’s moral purity increased a desire to engage in physical cleansing behaviors and the act of washing hands washed away one’s sins (i.e., reduced moral emotions such as disgust, regret, and guilt). Based on the results, Schnall et al. (2008) notably predicted that cleanliness would wash away other people’s sins. As predicted, they found that a cognitively activated concept of cleanliness and physical cleansing behaviors reduced the perceived severity of moral transgressions of others. Just as engaging in physical cleansing associated with moral cleanliness can result in less severe moral judgments, the bodily experience of finger prick that might be associated with moral guilt is expected to increase the severity of moral judgments of other people’s transgressions.

As noted above, we predicted that the sense of guilt might increase the sensitivity to physical prick and that physical prick would affect the moral judgments on others. In this article, we examined three studies that reveal a significant association between emotional- and physical prick. Participants were native Korean speakers who were familiar with the expression “It pricks my conscience” in the contexts of guilt. In Study 1, we investigated whether the manipulated sense of guilt would make participants less likely to engage in a traditional Korean remedy that involves finger prick (i.e., getting acupunctured) when they were assumed to be suffering from an upset stomach. In Study 2, we developed a situation where participants had to make a choice whether or not to tell the truth and observed their decision-making process. We then pricked their hands with an acupuncture device to examine whether the pain from the finger prick was stronger and if the prick felt deeper when participants had lied. In Study 3, we investigated whether the pricking sensation induced by the needle increased the severity of moral judgments as a downstream consequence of experiencing the embodied guilt.

## Study 1

In Study 1, we conducted a preliminary experiment to examine the association between prick of conscience and a temporary willingness to experience finger prick. If feelings of guilt are associated with the increased sensitivity to physical prick, participants who feel guilty may be less likely to experience the pain caused by the needle than the other types of pain. To test this hypothesis, we employed a traditional Korean treatment, which involves finger prick for an upset stomach (Stone et al., 2016). When Korean people have an upset stomach, they choose either to prick their fingers with the needle or take over-the-counter medication as a remedy.

Half of the participants were asked to recall past unethical acts, and the other half were asked to recall their ethical acts (i.e., between-subject design). We then asked all the participants to assume that they suffer from indigestion. Subsequently, they rated their willingness toward two different treatment methods: finger prick and medication (i.e., within-subject design). We predicted an interaction effect such that participants with increased feelings of guilt would be less likely to choose a method that involves finger prick, whereas those in the control group would not show any difference in their preferences over the two treatment methods.

### Method

#### Participants

Power analysis using G^∗^Power (Faul et al., 2007) suggested a minimum of 74 participants would be needed to detect an effect of moderate size (*f* = 0.25) of interaction effect at a power of 0.99 when conducting a two-way mixed ANOVA with two independent groups and two dependent variables. 90 undergraduate students (53 males, 37 females; mean age = 22.11 years, *SD* = 0.99 years) participated in this study, and they received a stationery product worth 3,000 Korean won (equivalent to the US $3) in return for their participation. This study was approved by the Research Ethics Committee of the Korea Military Academy. We obtained written informed consent from all participants prior to the study.

#### Procedure

Participants were informed that this study was to investigate the relationship between recalling ethical/unethical acts and the emotional state aroused by the memories. Participants were randomly assigned to two conditions (Recall: unethical vs. ethical), similar to other previous studies (e.g., Zhong and Liljenquist, 2006; Day and Bobocel, 2013). In the unethical condition, participants (*n* = 45) were asked to recall a situation where they had committed unethical acts. They were then instructed to describe past situations and note their feelings attached to the memories. To maintain confidentiality, participants were informed that they might jot down a few keywords that they had recalled if they wanted and were informed that those keywords would not be revealed. In the ethical condition, participants (*n* = 45) were asked to recall and describe their ethical acts in the past.

Immediately after priming, participants were asked to rate their guilty feelings on an Adapted Shame and Guilt Scale (ASGS; Hoblitzelle, 1987). We adopted a Korean version of ASGS (Nam, 2007). Among 30 items in total, we used 15 items that measured guilt for this study (i.e., “condemned,” “liable,” “wrong,” “unethical,” “guilty,” “chided,” “reproached,” “immoral,” “delinquent,” “unconscionable,” “wicked,” “criminal,” “indecent,” “unscrupulous,” “imprudent”; α = 0.96). The items were rated on a scale of 1 (not at all) to 5 (very much).

Participants were then asked to assume that they were suffering from indigestion at that moment. They were given two treatment options for alleviating the upset stomach; option one was to take a strong dose of medication, and option two was finger prick as part of the traditional Korean treatment (Stone et al., 2016). Participants rated how willing they were to choose each option to treat their imaginary stomachache on a scale of 1 (never willing) to 9 (very willing). These two treatment options were presented in random order and were expected to produce the same level of pain and anxiety. These options were tested in a pilot test with an independent sample of 18 undergraduate students, and we found that the two options were equally preferred to relieve indigestion without priming (pricking a fingertip: *M* = 6.28, *SD* = 1.57, taking a dose of medication: *M* = 6.39, *SD* = 1.75), *t*(17) = −0.36, *p* = 0.73, on a scale of 1 (not favor at all) to 9 (favor strongly). Finally, we obtained their demographics, and debriefing followed.

### Results and Discussion

**FIGURE 1:**
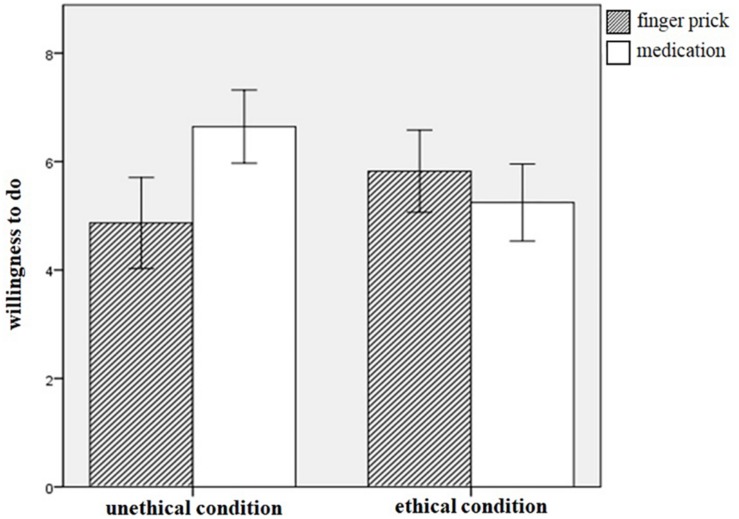
Willingness to prick a finger with a needle and to take a strong dose of medicine to treat indigestion across two conditions in Study 1. Error bars represent 95% CI.

Participants who recalled personal unethical acts (*M* = 3.82, *SD* = 0.84) reported heightened feelings of guilt than those who recalled ethical acts (*M* = 2.20, *SD* = 0.95), *t*(88) = 8.51, *p* < 0.001, *d* = 1.79. This result suggests that the priming was successful^1^. Next, we conducted a 2 (condition: recalling unethical act vs. ethical act) × 2 (option: pricking a finger with a needle vs. taking a strong dose of medication) two-way mixed ANOVA. Results indicated that a non-significant main effect for recall, *F*(1,88) = 0.34, *p* = 0.56, *r* = 0.06. The main effect of option was also not significant, *F*(1,88) = 2.76, *p* = 0.10, *r* = 0.17. Findings, however, were qualified by a significant interaction effect, *F*(1,88) = 10.62, *p* = 0.002, *r* = 0.33 (Figure 1). Specifically, the simple effect analysis showed that participants in the unethical condition were less likely to prick their fingers with the needle (*M* = 4.87, *SD* = 2.79) while it did not affect their choice of taking medication (*M* = 6.64, *SD* = 2.25), *F*(1,88) = 12.10, *p* = 0.001, *r* = 0.35. However, those in the ethical condition did not show any difference in their preferences over the two options (finger prick: *M* = 5.82, *SD* = 2.53, medication: *M* = 5.24, *SD* = 2.37), *F*(1,88) = 1.28, *p* = 0.26, *r* = 0.12.

As predicted, participants who recalled past unethical acts reported decreased interests in the treatment option with the finger prick as compared to taking a strong dose of medication as a treatment, whereas this pattern was not found among participants who recalled their ethical acts. The result of Study 1 substantiates the first hypothesis that the emotional prick of conscience is associated with the bodily sensation of physical prick.

It is notable that there was no significant difference in participants’ willingness to treat their imaginary symptom (i.e., the mean composite of pricking one’s finger and taking a strong dose of medication) between the unethical group (*M* = 5.76, *SE* = 0.27) and the ethical group (*M* = 5.53, *SE* = 0.27), *F*(1,88) = 0.34, *p* = 0.56. If guilty feelings make people more sensitive to pain in general, there should be the main effect of condition (i.e., unethical vs. ethical conditions) indicating lower mean scores in the unethical condition compared to those in the ethical condition. Accordingly, Study 1 rules out an alternative explanation that guilt makes people more reluctant to pain in general. Overall, this result suggests that prick of conscience decreases the likelihood of people experiencing the finger prick.

## Study 2

If Study 1 addressed the association between guilt-inducing memories and unwillingness to engage in the physical pain induced by the prick, Study 2 focused on exploring the links between feelings of guilt in the *status quo* and the finger prick. Specifically, we examined whether participants who told a lie would become more sensitive to the pricking stimulus than those who told the truth. Participants in the test condition were given a situation where they had to determine between telling a lie (i.e., the lie condition) and telling the truth (i.e., the truth condition), whereas participants in the control condition were not presented this situation. They were then pricked their hands with the needle three times. They rated how deep they sensed the needle and how painful it was. We expected that the guilty conscience induced by the act of lying would lead to a more sensitive reaction. To be specific, when pricked by the needle, we predicted that participants in the lie condition would sense the prick deeper and with more pain as compared to those in the truth- and the control condition. However, we did not have any specific prediction for the difference between the truth- and the control groups.

### Method

#### Participants

154 undergraduate students (72 males, 82 females; mean age = 20.58 years, *SD* = 1.14 years) participated in this study, and they received partial course credits and 3,000 Korean won (the US $3) in return for their participation. Power analysis using G^∗^Power (Faul et al., 2007) suggested a minimum of 137 participants would be needed to detect a large effect size (*f* = 0.40) at a power of 0.99 when conducting ANOVA with three independent groups.

#### Procedure

Study 2 was a correlational research design and consisted of two sessions. Instead of manipulating participants to feel guilty, we let participants decide whether or not to tell a lie so that they would be divided naturally into self-selected sub-groups (i.e., the lie group or truth group). To control potential variables that might affect the physical sensation of the needle prick, we asked a total of 144 participants to complete two assessments in the first session. First, we measured participants’ sensitivity to a sensory stimulus. Previous research indicated that somatosensory amplification was associated with pain perception (Lee et al., 2010; Ferentzi et al., 2017). Thus, we adopted the Somatosensory Amplification Scale (SSAS; Barsky et al., 1990) to evaluate the base level of participants’ sensitivity to sensory experiences prior to the experiment. The SSAS included 10 items on a five-point Likert scale (1 = not at all to 5 = very much; α = 0.72) and we used the Korean version of SSAS (Won and Shin, 1998).

In addition, Cohen et al. (2011) argued that people who were prone to feeling guilty were less likely to deceive others. Accordingly, we measured participants’ dispositional guilt with ASGS (Hoblitzelle, 1987) to explore the link between dispositional guilt and tendencies to lie as well as its impacts on the degree of sensitivity to the pricking stimulus. Similar to Study 1, 15 items were selected among 30 items in ASGS (Hoblitzelle, 1987). Participants were asked to rate to what extent they usually experienced feelings of guilt (1 = not at all to 5 = very much).

The second session was conducted a month after the first session. Participants were randomly assigned to a test group (*n* = 106) and a control group (*n* = 48). Participants in the test group had to contemplate over whether or not to tell the truth, whereas those in the control group did not have to. There were three laboratory rooms; in the first room, participants were greeted, in the second room they were primed depending on each condition, and finally in the third room they had the needle prick for three times.

Upon arrival, participants checked in the first room and each participant was escorted to the second room. In the second room, the participant was informed about the purpose of the study, where the research staff member deliberately mentioned the compensation. The prompt is followed.

“The experiment will be conducted in the next room (the third room) and will take about 10 min. We will give you 3,000 Korean won in return for your participation. By the way, you are very lucky because our research budget has been cut down recently that you are the last participant to receive 3,000 Korean won. From the person that comes right after you, he or she will be given only 1,000 Korean won. We are telling you this because we want you to take this study seriously. Please read the consent form and sign it for us.

When the staff member tried to leave the room to prepare for the next experiment, the guide suddenly entered into the room (on purpose) with an actor who pretended to be another participant. The guide then told the staff member that the actor came in earlier than expected. The staff member welcomed the actor, told the actor to wait for a minute, and left the room. Eventually, the participant and the actor were left together in the second room for about 3 min. The actor would look at the consent form for a while and asked the participant as below.

“Hello. I am participating in the study as well. Did you hear anything about the experiment from the staff? (Pointing the part in the consent form where it states remuneration) We may receive some money for participation. Have you heard about how much we would get paid?”

We wanted to pay special attention to how participants answered to the actor’s last question. After 3 min, the staff returned to the second room and took the participant to the next room (third room). The actor wrote down the participant’s responses on the note. Participants assigned to the control condition did not see the actor. They were escorted to the third room straight away after signing the consent form in the second room.

In the third room, participants were finger-pricked three times with an acupuncture device. The acupuncture device was designed to be clicked like a ball-point pen so that the experimenter could prick the participants with consistent strength and depth. We set the needle to cut the skin 2 mm deep. The experimenter then pricked the participants’ non-dominant hands three times. In the first trial, participants were asked to put their hands on the desk, with their palms facing downwards, and the experimenter pricked the center of the back of participants’ hands with the acupuncture device. For the second attempt, they were asked to put their hands with their palms facing upwards, and the experimenter pricked the center of the participants’ palms. Lastly, for the third trial, they were asked to do the same as the second attempt, and the experimenter pricked the middle of the participants’ wrists. After participants were pricked by the experimenter in each trial, they rated the perceived depth of the prick from the device on a continuous slider that ranged from 0 (not being pricked at all) to 10 (being pricked very deeply). Participants also scored how painful the prick was, using the same slider scale from 0 (not painful at all) to10 (very painful). These two questions were randomly presented to the participants. As for how deep participants sensed the needle prick on the back of their hands, palms, and wrists, the ratings were averaged (i.e., mean composite of prick; α = 0.75). Likewise, the ratings for perceived pains from each trial were also averaged (i.e., mean composite of pain; α = 0.75).

Participants in the test group were then asked to answer five more questions: “Did you remember the participant who was waiting for the experiment with you in the previous room?,” “Did you remember the question that the participant asked you?,” “How did you respond to the question?,” “Do you think that you lied to the participant?” Finally, they were asked to rate how much they felt guilty about their response on a scale of 1 (not guilty at all) to 9 (very guilty). Lastly, participants were thoroughly debriefed and provided the compensation (3,000 Korean won). Participants in the control condition were also given 3,000 Korean won.

### Results and Discussion

We first divided the participants in the test group into two subgroups; in one subgroup, participants told a lie and in another, they told the truth. It was based on three criteria – the experimenter’s judgment based on the participant’s answers recorded by the actor, the actor’s judgments, and the participants’ reports. Most of the participants were easily sorted to one of two groups (i.e., the lie group and truth group) as all three judgments coincided with each other. Responses that were considered to be truth included “I was told that I would receive 3,000 Korean won, but it will be 1,000 won for you because they said they had to cut down the budget and could not afford to pay 3,000 Korean won anymore.” or “3,000 Korean won.” On the other hand, participants who said, “I haven’t heard about the reward.,” “I don’t know about the compensation.,” or “1,000 Korean won.” were categorized as the lie group. However, some participants were difficult to be categorized as three judgments did not correspond to each other or even participants themselves could not identify if they lied or not. For example, their answers were, “The staff will directly inform you about the reward.” or “I think it’s not appropriate for me to tell you about the reward.” Thus, those in these cases (i.e., 11 participants) were excluded from the analysis. In addition, it was revealed in the debriefing that three participants had doubted the actor’s questioning. Thus, these three participants were also excluded from the analysis.

In consequence, 54 participants were classified into the lie group, 38 participants to the truth group, and 48 participants to the control group. Participants in the lie group (*M* = 4.24, *SD* = 2.94) reported that they felt more guilty at the moment of answering to the actor’s question about the pay than those in the truth group (*M* = 1.71, *SD* = 2.29), *t*(90) = 4.44, *p* < 0.001, *d* = 0.95. We then tested whether the dispositional guilt predicted the likelihood of lying, using logistic regression. Participants with higher levels of dispositional guilt were significantly more inclined to lie (*B* = 0.96, *SE* = 0.46, Wald χ^2^ = 4.40, *p* = 0.036, odds ratio = 2.61, 95% CI for odds ratio [1.07, 6.38]. This result rebutted Cohen et al. (2011)’s results that individuals with higher scores on guilt would become less deceptive.

Next, a one-way ANOVA analysis was conducted to determine if dispositional sensitivity to sensory experiences were varied among groups. Results indicated a non-significant effect between the lie group (*M* = 2.95, *SD* = 0.47), the truth group (*M* = 3.07, *SD* = 0.46), and control group (*M* = 2.96, *SD* = 0.70), *F*(2,137) = 0.83^2^, *p* = 0.54, ω^2^ = −0.01.

To test whether participants in the lie group felt the prick deeper than those in the truth or control groups, we conducted a one-way ANOVA analysis. As predicted, there was a significant difference between groups, *F*(2,137) = 11.34, *p* < 0.001, indicating a large effect size, ω^2^ = 0.13. Scheffé *post hoc* test showed that participants in the lie group (*M* = 4.95, *SD* = 1.28) sensed the needle prick deeper than those in the truth group (*M* = 3.86, *SD* = 1.38), *p* = 0.001 and the control group (*M* = 3.80, *SD* = 1.44), *p* < 0.001. There was no significant difference between the truth group and the control group, *p* = 0.98^3^. When tested for each condition (i.e., back of one’s hand, palm, and wrist), the same pattern was found in the results (see Table 1).

**TABLE 1:** Descriptive statistics and significance tests in Study 2.

**Conditions**	**Lie (*n* = 54)**	**Truth (*n* = 38)**	**Control (*n* = 48)**	***F***	**Scheffé**
			
	***M*(*SD*)**	***M*(*SD*)**	***M*(*SD*)**		
**Prick**					
Mean composite	4.95 (1.28)	3.86 (1.38)	3.80 (1.44)	11.34***	a > b = c
Back of hand	3.92 (1.68)	2.81 (1.55)	2.88 (1.80)	6.64**	a > b = c
Palm	5.85 (1.84)	4.83 (1.86)	4.85 (1.72)	5.22**	a > b = c
Wrist	5.08 (1.44)	3.95 (1.66)	3.66 (1.79)	10.83***	a > b = c
**Pain**					
Mean composite	4.08 (1.49)	3.26 (1.11)	3.27 (1.49)	5.71**	a > b = c
Back of hand	2.71 (1.65)	1.81 (1.16)	2.07 (1.63)	4.36*	a > b
Palm	5.21 (2.19)	4.30 (1.53)	4.23 (1.92)	3.99*	a > c
Wrist	4.33 (1.91)	3.65 (1.62)	3.51 (1.92)	2.88	a = b = c

Prick represents how deep participants sensed being pricked and response scale ranged from 0 (not being pricked at all) to 10 (being pricked very strongly). Pain means how painful participants felt and the scale ranged from 0 (not painful at all) to 10 (very painful). a = lie group, b = truth group, c = control group. ***p < 0.001, **p < 0.01, *p < 0.05.

In a similar vein, we examined whether the level of pain (i.e., mean composite of pain) appeared different among groups. There was a significant group difference, *F*(2,137) = 5.71, *p* = 0.004, ω^2^ = 0.25, and the Scheffé test indicated that the lie group (*M* = 4.08, *SD* = 1.49) felt more pain when pricked by the device than the truth group (*M* = 3.26, *SD* = 1.11), *p* = 0.02, and the control group (*M* = 3.27, *SD* = 1.49), *p* = 0.02. The truth group and the control group did not differ from each other, *p* = 0.99^4^. The one-way ANOVA test for each trial yielded slightly different results and yet, we found a tendency regardless. The lie group felt most painful while the truth group and the control group felt relatively less painful (see Table 1).

In sum, Study 2 substantiates our hypothesis that prick of conscience would be related to increased sensitivity in the experience of physical prick. To be specific, participants whose conscience pricked them due to telling lies right before the experiment (i.e., lie group) sensed the prick deeper and felt more pain than the other participants in the truth group and the control group.

Study 2 reaffirms and expands the results from Study 1 in that the significant association between prick of conscience and physical prick was found in the real setting. Considering that participants in the truth group and the control group had the same base level of sensation for the needle prick, Study 2 suggests that perceived feelings of guilt (i.e., lying) does have an influence on the physical sensation caused by pricking.

## Study 3

Studies 1 and 2 showed that prick of conscience induced by recalling and engaging in unethical acts could be embodied in the physical prick. As discussed earlier, Schnall et al. (2008) stated that physical cleansing reduces the severity of moral judgments. They predicted that people would perceive moral transgression less negatively after physically cleansing themselves. As a result, they found that physical cleanliness was cognitively activated by a scrambled-sentences task (Study 1) and the act of physical cleansing (i.e., hand washing, Study 2) made moral judgments on other’s misdeeds less severe.

Given that physical cleansing reduces perceived wrongness of moral transgression, the physical prick associated with a sense of moral guilt may increase the severity of moral judgments on other’s transgressions. Thus, we conducted Study 3 to expand the findings from Studies 1 and 2 by examining the downstream impacts of the embodied guilt on moral judgments. We constructed a hypothesis that participants who were pricked by the needle would make more severe moral judgments than those who were not.

### Method

#### Participants

Power analysis using G^∗^Power (Faul et al., 2007) suggested a minimum of 137 participants would be needed to detect an effect of large size (*f* = 0.40) at a power of 0.99 when conducting the one-way ANOVA test with three independent groups. In this study, 137 undergraduate students (68 males, 69 females; mean age = 21.31, *SD* = 1.64 years) participated and received stationery products worth 3,000 Korean won in return for their participation.

#### Procedure

Participants were informed about the purpose of the study that examined the relationship between physical stimulus and pain. They were randomly assigned to one of the three conditions – the strong prick, the weak prick, and no prick (control group). In two conditions that involved the needle prick, participants’ hands were pricked three times and were asked to rate their sensation as to how deep they were pricked and how painful it was. Participants in the strong prick condition (*n* = 47) were pricked 3 mm deep and those in the weak prick condition (*n* = 46) were pricked 1 mm deep. Participants in the control group (*n* = 44) were not pricked.

After pricking, participants were informed that they would participate in a separate experiment on moral judgments. They were instructed to judge several moral dilemmas. Moore et al. (2008) created 24 critical dilemmas and 14 filler dilemmas based on existing studies (i.e., Greene et al., 2001, 2004). Critical dilemmas made participants contemplate over a scenario where they would have to kill one person to save many others. Filler scenarios included similar moral dilemmas, however, they did not involve killing people. Focusing on minor moral issues in the present study, we utilized eight moral dilemma situations related to stealing, lying, and being dishonest with the fillers: “Been Caught Stealing,” “Taxes,” “Stock Tip,” “Plasma Screen,” “Resume,” “Illegal Lunch,” “Employee Morale,” and “Insurance Fraud.” Participants were asked to rate whether the protagonist’s behaviors were appropriate in each scenario on a scale of 1 (perfectly okay) to 9 (extremely wrong). To rule out the influence of emotional reactions from the priming, we asked participants to take another assessment called, “Positive and Negative Affect Schedule (PANAS; Watson et al., 1988).” We used the Korean version of PANAS (Park and Lee, 2016). Finally, they reported their age and sex, and were debriefed.

### Results and Discussion

Participants in the strong (3 mm) prick condition (*M* = 5.34, *SD* = 1.65) reported that they sensed the needle prick deeper than those in the weak (1 mm) prick condition (*M* = 3.55, *SD* = 1.83), *t*(91) = 4.94, *p* < 0.001, *d* = 1.03. They (*M* = 4.19, *SD* = 1.57) also reported more pain to the needle prick compared to participants in the weak prick condition (*M* = 2.61, *SD* = 1.72), *t*(91) = 4.62, *p* < 0.001, *d* = 0.96. Next, we tested whether the priming affected the emotion ratings at the end of the experiment by conducting the one-way ANOVA analysis. The results showed that there was no statistically significant difference in the scores for both positive affect, *F*(2,134) = 1.57, *p* = 0.21, ω^2^ = 0.01, and negative affect, *F*(2,134) = 0.17, *p* = 0.85, ω^2^ = −0.01, which leads to a conclusion that the priming did not appear to induce any positive or negative emotional reactions to the participants.

We then computed the mean composite of all eight moral dilemmas and examined whether the needle prick increased the severity of moral judgments. The one-way ANOVA analysis on the composites showed the effect of condition, *F*(2,134) = 23.44, *p* < 0.001, ω^2^ = 0.14. There was no significant difference in moral judgments between the strong- and the weak conditions, *p* = 0.90. However, participants in the strong prick condition appeared to have more negative moral judgments than the control group (*M* = 5.75, *SD* = 1.61), *p* < 0.001 and so did the weak prick condition, *p* = 0.001. The pattern of results was consistent across the scenarios (see Table 2).

**TABLE 2:** Means ratings for moral vignettes in Study 3.

**Condition**	**Strong prick (*n* = 47)**	**Weak prick (*n* = 46)**	**Control (*n* = 44)**	***F***	**Scheffé**
			
	***M*(*SD*)**	***M*(*SD*)**	***M*(*SD*)**		
Mean composite	7.07 (1.09)	6.93 (1.45)	5.75 (1.61)	12.06***	a = b > c
Been caught stealing	8.40 (1.83)	8.20 (1.64)	6.36 (2.13)	16.17***	a = b > c
Taxes	8.94 (1.26)	8.41 (2.15)	8.09 (1.12)	3.34*	a = b, a > c
Stock tip	7.02 (1.75)	6.65 (2.02)	5.89 (2.40)	3.53*	a = b, a > c
Plasma screen	8.83 (1.58)	8.98 (1.41)	7.64 (1.62)	10.28***	a = b > c
Resume	4.43 (2.04)	4.67 (2.36)	3.14 (2.49)	5.77**	a = b > c
Employee morale	6.32 (2.03)	6.02 (2.10)	4.61 (2.55)	7.46**	a = b > c
Insurance fraud	6.28 (2.29)	6.46 (2.43)	5.05 (2.81)	4.18*	a = b, b > c
Illegal lunch	6.32 (2.42)	6.07 (2.53)	5.25 (2.60)	2.22	a = b = c

Response scale ranged from 0 (perfectly OK) to 9 (extremely wrong). Mean Composite represents the mean of all eight moral vignettes. a = Strong prick condition. b = Weak prick condition. c = control condition. ***p < 0.001, **p < 0.01, *p < 0.05.

Study 3 demonstrated that needle prick increased the severity of moral judgments. Particularly, it is important to note that the depth of the needle (i.e., 3 and 1 mm) did not have a major effect on participants’ moral judgments but the pain induced by the needle prick did. Taken together, the data from Study 3 are consistent with our hypotheses that prick of conscience is associated with the sensation of the physical prick.

## General Discussion

This study was conducted to investigate whether prick of conscience would be grounded in bodily experiences of physical prick (e.g., a needle prick), using a sample of Korean participants who were familiar with the metaphorical expression “It pricks my conscience.” The results of the study lent support to our hypothesis that prick of conscience is associated with the physical sensation of pricking. Participants who recalled unethical acts (Study 1) and who lied (Study 2) appeared to become more sensitive to the needle prick than those who did not. In addition, participants who had the needle prick made more severe moral judgments than participants in the control condition (Study 3).

This study also provides several implications. First, the findings of the present study propose that metaphors do not only convey linguistic connotations but also plays a significant role in social cognition (Zhong and Leonardelli, 2008; Landau et al., 2010). Although a rich body of literature has identified embodied metaphors in words, very few studies have measured embodied metaphors in idioms. We suggest that “prick of conscience” would be regarded as another embodied metaphor, which broadens the understanding of the relationship between language and social cognition.

Second, the present study is noteworthy in that it is the first study to highlight the connection between embodied guilt and a sense of prick in South Korea. “It pricks my conscience” is a widely used expression among Koreans in the context of guilt or remorse. As Santana and de Vega (2011) illustrate how metaphors allow people to understand abstract concepts represented in the sensory-motor experiences, our studies of “prick of conscience” demonstrate that this metaphor can be experienced physically. Anderson (2003) also stresses the importance of a dynamic interaction between the human brain and cultural contexts when it comes to an individual’s embodied social cognition. This is also congruent with Leung et al. (2011)’s proposal of “embodied cultural cognition,” which indicates that body-mind linkages are not randomly formed but derived from the meanings informed by the socio-cultural contexts, such as cultural imperatives, values, and habits. Guilt is a universal emotion that people feel when they commit unethical acts or violate moral standards. However, the way that guilty feelings are expressed can vary. In Western culture, it is common to say, “I feel guilty,” whereas in Asian culture, especially in South Korea, people use the metaphor, “It pricks my conscience.” to articulate their guilty feelings. Accordingly, this metaphor can be deemed as culturally driven, although the current study cannot provide explanations for the role of cultural aspect in the associative link between emotional and physical prick.

Third, it is worth highlighting how our findings differ from those in earlier studies on the effect of experiencing physical pain after recalling or engaging in unethical acts. Previous research has been focused on self-punishment as a sign of remorse (Bastian et al., 2011; Nelissen, 2012; Inbar et al., 2013). When people feel guilty but have no opportunity to recompense, they tend to punish themselves to get rid of themselves of guilt. This tendency is called the “Dobby Effect” by Nelissen and Zeelenberg (2009). Nelissen (2012) contends that people are more willing to punish themselves with electrical shocks if the person they feel guilty for is presented in the same room. By contrast, they were inclined to punish themselves less intensively when they were alone. It also aligns with Bastian et al. (2011) conclusion that people who recalled personal unethical acts would hold their hands in the ice bucket longer and would rate the experience more painful than participants without the priming. These findings suggest that guilt-induced self-punishment serves as an atonement for sins, and thus, guilty people are more motivated to inflict physical pain to themselves. On the contrary, this paper focuses on punishment by others in that we made participants be pricked by others rather than pricking themselves with the needle. In addition, although participants in Study 1 rated the willingness to prick their fingers with the needle, finger prick in Study 1 was a therapeutic way to treat indigestion rather than self-punishment. Thus, the present study makes a positive contribution to understanding the source of physical pain in managing guilt. In other words, guilt can be eliminated to some extent by experiencing physical pain through self-punishment, however, the physical pain inflicted by others is not related to atonement, but only worsens the sense of moral purity.

Fourth, the current research contributes to the impacts of the absence of guilt on the bodily sensation. The results of Study 2 revealed that there was no significant effect of telling the truth on the sensation of being pricked. When participants told a lie (the lie condition), they reported higher sensitivity to the needle prick compared to participants who told the truth (the truth condition) or said nothing (the control condition). However, there were no differences in the sensation of the needle prick between the truth and the control conditions. Consistent with the results, feelings of guilt that participants felt when they replied to the actor’s question was not correlated with how deep they sensed the prick, *r* = 0.14, *p* = 0.20, and how painful they felt, *r* = 0.12, *p* = 0.27. These findings were consistent with the result of Day and Bobocel (2013) study that examined the relationship between guilt and subjective body weight. They found that unethical acts made participants feel heavier than they usually do, whereas ethical acts did not make participants feel any lighter. That is, there was no difference in perceived weights between participants who recalled personal ethical acts and those who did not recall any memories at all. Thus, these findings suggest that a lack of guilt does not affect the bodily sensation in relation to the guilt.

Fifth, participants’ moral judgments were influenced by whether or not they had the needle prick (i.e., 3 and 1 mm) rather than the depth of the prick in Study 3. This finding is in line with the previous research on how hand washing reduces moral taint such as physical disgust, regret, or guilt (Zhong and Liljenquist, 2006) and makes moral judgments about others more severe (Schnall et al., 2008). They also found that a mere act of washing was attributed to moral purity, not how many times people wash their hands to get rid of moral transgressions. Likewise, the current findings suggest that the prick itself influences participants’ moral judgments on others regardless of the depth of the needle prick.

Sixth, we should note previous studies that examined the relationship between physical cleanliness and moral judgments. In Study 3, we found that the bodily experience of physical prick led to more severe moral judgments as a consequence of embodied guilt. This is congruent with Schnall et al. (2008) study that physical cleansing after feeling disgusted from a movie reduces the severity of moral judgments. However, one notable work reveals a contradictory finding that physical cleanliness leads to harsher moral judgments (Zhong et al., 2010). Researchers point out that the effect of cleanliness or dirtiness is context-sensitive, which means that physical cleansing after a disgusting experience is different from the cleansing behavior without any pre-experience of disgust (Zhong et al., 2010; Lee and Schwarz, 2011). To be specific, they argue that removing dirty residues (i.e., physical cleansing) from one’s mind after watching a disturbing film presumably attenuates disgust and hence makes the perception of transgression on others less aversive. Similarly, feeling cleaner after engaging in physical cleansing without experiencing disgust leads to moral superiority and therefore, renders harsher judgments on others’ immoral acts. In this respect, it is possible that physical prick leads to moral inferiority in Study 3, as we made participants pricked by the needle without any pre-inducement of feelings. In contrast to the explanations by the previous authors, however, the results of Study 3 indicated that physical prick resulted in harsher judgments. We argue that this inconsistency is derived from the different nature of the moral vignettes used in the studies. In Zhong et al. (2010) study, participants judged contested social issues chosen by the authors including alcoholic, casual sex, homosexuality and many others. However, as Zhong et al. (2010) mentioned, participants were asked to make moral judgments on social issues with ambiguous moral implications. Similarly, Schnall et al. (2008) used six moral vignettes of which was called the “Trolley” problem that made participants decide whether to switch the track of a trolley to kill one worker in order to save five others. However, these scenarios did not represent obvious moral transgressions. Indeed, the effect of physical cleanliness on the ratings of each dilemma showed inconsistent results between Studies 1 and 2 in their research. In contrast to their studies, our research used moral scenarios where the protagonist commits obvious moral transgressions such as lying or stealing. We believe this is the strength of our study in that the findings highlight the importance of using clear moral scenarios for priming. Future studies might consider investigating the nuance of the relationship between embodied guilt and moral judgments with more relevant moral scenarios.

Seventh, although it was not our main interests of the present study, it is important to note the comparison between Study 2 of our research and Study 2 of Cohen et al. (2011)’s experiment. Both studies followed similar procedures where participants had to decide whether or not to lie. Interestingly, our results contradict the results of Cohen et al. (2011). 54 participants (59%) lied and 38 (41%) told the truth in our study, whereas 23 (32%) lied and 49 (68%) told the truth in Cohen et al. (2011)’s experiment. Furthermore, participants who lied had a stronger dispositional trait to feel guilty compared to those who told the truth in the current study, and participants with a stronger inclination to feel guilty are less likely to lie in Cohen et al. (2011)’s study. Two studies provided different motivational contexts where in Cohen et al. (2011)’s study, deceiving other participants was directly related to the compensation while it did not affect the reward in our study. We believe that such conflicting results were attributed to the participants’ different cultures. Guilt is differently conceptualized across cultures (Bedford and Hwang, 2003; Bedford, 2004; Anolli and Pascucci, 2005; Wong and Tsai, 2007). In western culture, individuals feel guilty when they fail to embrace their authentic selves (Spicer, 2011). On the other hand, the sense of duty and obligations to significant others matter hugely in eastern culture (i.e., Chinese culture; Bedford, 2004). Therefore, the seemingly disparate results imply that guilt may be manifested differently across cultures (Tangney et al., 1989). Further research will be needed to fully disentangle the relationship between dispositional guilt and lie.

Finally, it is worth noting that some recent attempts to replicate the existing findings in the embodied cognition have failed (e.g., Johnson et al., 2014; Lynott et al., 2014). Researchers criticized that underpowered studies contribute to low success rates of replication studies (Perugini et al., 2014; Anderson et al., 2017). In Study 1 (*n* = 90), we found a significant interaction effect, *F*(1,88) = 10.62, *p* = 0.002, yielding a medium effect size, *r* = 0.33 with power of 90%. In Study 2 (*n* = 140), the results showed a significant difference in the perception of vividness of being pricked among groups, *F*(2,137) = 11.34, *p* < 0.001, indicating a large effect size, ω^2^ = 0.13 with 99% power. We also found that the perception of pain varied among groups, *F*(2,137) = 5.71, *p* = 0.004, yielding a large effect size, ω^2^ = 0.25, and 86% power. In Study 3 (*n* = 137), the results indicated a significant difference in moral judgments (i.e., mean composite of all eight dilemmas) among groups, *F*(2,134) = 23.44, *p* < 0.001, with a large effect size, ω^2^ = 0.14, and 99% power. Taken together, the effect size and statistical power observed in our research indicate that our study is not underpowered and therefore, stand statistically strong despite the replication crisis in the field.

### Limitations

Although the findings and the implications of our studies are compelling, there are some limitations. First, in Study 1, we found that when we asked participants who recalled past transgressions to rate how willing they were to prick their fingers with the needle or to take a strong dose of medication, the rating for the needle prick was higher than the score for taking medication, whereas the ratings for both options were not significantly different to the participants who recalled ethical acts. Although the results were consistent with our hypothesis, an additional simple effect analysis based on each option indicated that willingness for finger prick was not different between participants in the unethical condition (*M* = 4.87, *SD* = 2.79) and those in the ethical condition (*M* = 5.82, *SD* = 2.53), *F*(1,88) = 2.90, *p* = 0.09, *r* = 0.42. On the other hand, the willingness to take a strong dose of medication was different between conditions (unethical condition: *M* = 6.64, *SD* = 2.25, ethical condition: *M* = 5.24, *SD* = 2.37), *F*(1,88) = 8.28, *p* = 0.005, *r* = 0.29). Thus, the results may suggest that feelings of guilt may increase the willingness to take medication in lieu of pricking the finger. However, we note that the difference in willingness for finger prick between the two groups is marginally significant, *p* = 0.09, yielding a larger effect size, *r* = 0.42 than willingness for taking strong medication, *r* = 0.29. Although the pilot test with 18 undergraduate students confirmed that the two treatments were favored almost equally, we did not measure nor control participants’ pre-existing preferences for the main study. Thus, another avenue for future research is to consider measuring individuals’ preferences for each treatment methods prior to the priming.

Second, the design of Study 2 was not experimental but correlational. We did not manipulate participants’ decisions to tell a lie or not. Rather, they made their own choices. Accordingly, the results could not draw causal relationships between the act of lying (independent variable) and the physical sensation of the needle prick (dependent variable). Thus, given the descriptive nature of non-experimental research, Study 2 could not address third-variable problems, leaving other interpretations for the results. Although we measured dispositional sensitivity to sensory experiences and found that there was no significant difference across conditions, we did not manipulate participants’ decisions to engage in guilt-induced behaviors because we were aware of the possibility that participants might attribute the act of lying to others, in this case the experimenter, and accordingly, might not feel guilty in a genuine sense. Therefore, we welcome future research that extends our work in investigating the links between the guilty conscience and physical sensation of the needle prick with relevant experimental designs.

Third, previous research suggests a two-way relationship between mind and body such that feelings of guilt increase a desire to engage in physical cleansing behaviors while physical cleansing reduces guilty emotions (Zhong and Liljenquist, 2006). In the current research, Studies 1 and 2 focused on the effects of guilt in the sensation of physical prick, whereas Study 3 examined the effects of the physical prick on moral judgments of others’ misbehaviors. However, Study 3 did not represent the reversed effect of Studies 1 and 2 because we assessed how participants perceived moral transgressions on others rather than their moral self-images. Thus, the results of Study 3 indicate subsequent effects of experiencing the embodied metaphor, “prick of conscience.” Future research can inquire the effects of physical prick on moral self-judgments with relevant experimental designs.

Finally, our sample is limited to Korean populations who are accustomed to the metaphor “It pricks my conscience,” and thus, our studies might not be applicable to other populations with different cultural backgrounds. To our knowledge, there is no other countries except for South Korea that articulate feelings of guilt through sensations such as pricking or piercing. Although Japanese and Chinese populations share similar cultural values and norms with Koreans, they do not have the expression “prick of conscience” in their languages to reflect feelings of guilt. Due to within and between cultural variations, it is most likely that other researchers, even though they share similar Asian culture, will yield results that might be different from what we have found. Several avenues for future studies include to what extent this linguistic expression of “prick of conscience” would be valid in cross-cultural studies since this term is considered to be culturally- and linguistically bounded to Koreans.

## Conclusion

Overall, this research provides evidence that prick of conscience is not just a linguistic metaphor but it evokes both emotional and physical responses. If *The Prick of Conscience* is the most popular poem in Middle English reflecting religious aspects of washing away the sins, “prick of conscience” in the Korean metaphor can be interpreted as a manifestation of cultural language and social context.

## Data Availability Statement

The datasets generated for this study are available on request to the corresponding author.

## Ethics Statement

This study was carried out in accordance with the recommendations of the American Psychological Association, with written informed consent from all participants. All participants gave written informed consent in accordance with the Declaration of Helsinki. The protocol was approved by the Research Ethics Committee of the Korea Military Academy.

## Author Contributions

XK and JL conceived, designed, and conducted the study. XK wrote the first draft of the manuscript. JL performed the statistical analysis. HL contributed to the interpretation of the results. All authors revised the manuscript, and read and approved the final manuscript.

## Conflict of Interest

The authors declare that the research was conducted in the absence of any commercial or financial relationships that could be construed as a potential conflict of interest.

## Footnotes

1 The unethical acts that participants shared included lying (*n* = 20), covering one’s mistake (*n* = 5), stealing (*n* = 3), betraying friends (*n* = 2), cheating on dating partner (*n* = 1), violating regulation (*n* = 1), discriminating other sexually (*n* = 1), and unidentified (i.e., reporting just key words, *n* = 12).

2 Levene’s test indicated unequal variances, *F*(2,137) = 6.44, *p* = 0.002, we report Welch’s F.

3 When conducting ANCOVA where the score of SSAS (Barsky et al., 1990) served as the covariate, the results showed same pattern, *F*(2,136) = 11.28, *p* < 0.001, ω^2^ = 0.11, indicating the lie felt the prick deeper than the truth group, *p* < 0.001, and control group, *p* < 0.001. The score of SSAS was not associated with the sensation of being pricked, *F*(1,136) = 0.03, *p* = 0.86.

4 When controlling for the score of SSAS, there was also a significant difference between groups, *F*(2,136) = 5.90, *p* = 0.004, ω^2^ = 0.10. The lie group felt more pain than the truth group, *p* = 0.005, and control group, *p* = 0.004, while SSAS score did not affect the feeling of pain, *F*(1,136) = 1.12, *p* = 0.29.
